# A review of argasid ticks and associated pathogens of China

**DOI:** 10.3389/fvets.2022.865664

**Published:** 2022-07-26

**Authors:** Ze Chen, Jingze Liu

**Affiliations:** Hebei Key Laboratory of Animal Physiology, Biochemistry and Molecular Biology, College of Life Sciences, Hebei Normal University, Shijiazhuang, China

**Keywords:** Argasidae, host and distribution, molecular characters, tick-borne pathogens, China

## Abstract

It has been recorded 221 species of soft ticks in the world. However, the classification system of Argasidae is still unclear with nearly two-third controversial species in genus level. Therefore, comprehensive research is still necessary. In 2016, Wen and Chen overviewed the valid species of soft ticks in China for the first time. Up to now, the soft tick fauna of China remains poorly known. Although several studies have been undertaken, the information regarding soft ticks and associated diseases are fragmentary. To facilitate the future study of this group, the scattered information on soft ticks of China is herein synthesized. Toward the end of 2021, 15 valid species of argasid ticks have been reported, of these, 9 species (60%) including *Argas beijingensis, A. japonicus, A. persicus, A. sinensis, A. vespertilionis, A. vulgaris, Ornithodoros lahorensis, O. tartakovskyi*, and *O. papillipes* have been recorded biting humans. *Argas persicus* is the most common species, and its borne pathogens are widely investigated, while most other argasid ticks are not sufficiently studied in China. Here, we summarize detailed information regarding hosts, geographical distribution, molecular data, and vector roles of argasid ticks in China.

## Introduction

Ticks are obligate hematophagous ectoparasites of a wide variety of mammals, birds, reptiles, and amphibians. They cause direct injuries by blood-sucking and are important vectors of a large variety of human, domestic, and wild-animal pathogens, including viruses, bacteria, and protozoans, which can damage to livestock production and human health (1). Tick species can be grouped into three current families (Argasidae, Ixodidae, and Nuttalliellidae) and one extinct family (Deinocrotonidae) (1, 2). Argasidae is second to Ixodidae with regard to the number of species. However, there is widespread disagreement concerning the taxonomy above the species level (i.e., subgenus and genus) in this family, with nearly two-third controversial species (3). According to various schools of scientific thought, the following five classification systems have been proposed for Argasidae: The American school of acarologists (4, 5), the French school (6, 7), the Soviet school (8–10), the cladistic scheme of Klompen and Oliver (11), and, most recently, a molecular system of classification by Mans and colleagues (12). The classification systems of American and Soviet schools are based on unique morphological characters, which are determined by the degree of phenetic differentiation, without reflecting the evolutionary history. The classification system of cladistic school is based on morphology and biology, and was first proposed from a phylogenetic perspective. The French school only proposes a simple list of taxonomic rank, in which the taxa are not supported by morphological or biological characters. Burger et al. (13) first tested the genus–level classification of soft ticks by using mitochondrial genome and nuclear rRNA sequences. Their analyses strongly supported a clade of neotropical species within the subfamily Ornithodorinae, which included species from two genera, *Antricola* and *Nothoaspis*, and two subgenera, *Ornithodoros* (*Alectorobius*) and *Ornithodoros* (*Subparmatus*). Additionally, their analysis strongly supported a clade called *Ornithodoros sensu stricto* consisting of *O. savignyi* and four other *Ornithodoros* species (*O. brasiliensis, O. moubata, O. porcinus*, and *O. rostratus*) (13). Mans et al. (12) first proposed a molecular classification system for soft ticks based on the mitochondrial genome and nuclear sequence data. This classification system corresponds broadly with that of Klompen and Oliver (11), in which *Carios* and *Chiropterargas* were included in the subfamily Ornithodorinae, and *Alveonasus* in the subfamily *Argasinae*. There were also modifications made to several genera and subgenera. For example, the taxonomic status of *Ogadenus, Secretargas, Proknekalia, Alveonasus*, and *Chiropterargas* suggested as subgenera by Klompen and Oliver (11) were all promoted to the genus level by Mans et al. (12). Additionally, Mans et al. (12) established a new genus, *Navis*. This molecular classification system has essential reference significance. Later, Mans et al. (14) modified this classification scheme after analyzing the phylogenetic status of the bat tick *Argas vespertilionis* (Latreille) (*Carios vespertilionis*), and suggested that the subfamily Argasinae should be divided into six genera: *Alveonasus, Argas, Navis, Ogadenus, Proknekalia*, and *Secretargas*. The subfamily Ornithodorinae contains nine genera: *Alectorobius, Antricola, Carios, Chiropterargas, Nothoaspis, Ornithodoros, Otobius, Reticulinasus*, and *Subparmatus* (12, 14). This represents significant progress in the systematic classification of soft ticks. However, the further studies involving more controversial species and species from understudied regions should be conducted.

China is a country whose argasid fauna is poorly known (only 15 species reported thus far) (15, 16). In China, studies on ticks prior to the 1960s are scarce and not systematically documented. According to Li (17), the earliest research on soft ticks can be traced back to 1929, when Faust found an *Argas* sp. on domestic dogs in China. Later, Feng Lanzhou began to study the development of *Borrelia duttoni* in *O. moubata* collected abroad. In 1951, Feng and Huang collected *A. persicus* (Oken) from Shanxi province (17). Since then, research on soft ticks in China has been gradually developing, and includes case reports, morphological descriptions, biological characters, pathogens, and studies on the protein composition and karyotype characters of ticks (18–50). Wen and Chen (15) reviewed valid species of soft ticks for the first time (15). Toward the end of 2015, they listed valid argasid names of the world and China, and proposed a Chinese scientific term for each valid species and genus. Chen and Yang (51) published a monograph named “Systematics and taxonomy of Ixodida,” in which argasid ticks from China were systematically redescribed. Over the past two decades, we have witnessed the emergence and re-emergence of tick-borne diseases. However, systematic surveys of soft ticks and associated pathogens still lack in China. Here, we reviewed literature on soft ticks published in Chinese, English, Russian, and Japanese to provide a detailed summary of argasid ticks and associated pathogens in China.

## Argasid ticks and associated pathogen in fauna of china

As previously described, the classification system for soft ticks requires improvement. With further studies on more controversial species and the application of integrated methods, the taxonomic status of some tick species or groups is likely to change in the future. To prevent confusion in species names caused by constant changes, we followed Guglielmone et al. (3) and temporarily adopted the genus-level classification of Argasidae proposed by Hoogstraal (5) throughout this article. With regard to the nomenclature of the tick hosts, we place the genus name after the common name, except for the hosts identified to species level by authors. Some host species might have been misidentified; however, to avoid missing information, we quote the original name reported in the literature.

Currently, the argasid tick fauna of China consists of 15 species from two genera, *Argas* (10 species) and *Ornithodoros* (5 species). An overview of each soft tick species in China is presented below. Additionally, information on the deposition of type material of tick species first discovered in China is presented in this study. The administrative and biogeographical divisions of China are based on Chen et al. (52).

### *Argas assimilis* Teng & Song, 1983

This species was first described in Jiangxi Province of China (45). The meaning of the specific name “*assimilis*” is “similar and closely resembling” (45).

#### Type depositories

Institute of Zoology, Chinese Academy of Sciences (IZAS) (holotype ♀, allotype ♂, paratypes 2♀♀ 2♂♂ and 1 nymph); Jiangxi Medical College of China (paratypes 4♀♀).

#### Local distribution

Oriental Region (Jiangxi, Guizhou) (45, 51–54).

#### Natural host

Passeriformes: swallow (*Hirundo daurica japonica*) (45, 51–54).

#### Habitats

Swallows' nest.

#### Molecular data

No record.

#### Tick-borne pathogens

No record from China.

#### Remarks

This species is closely related to *A. japonicus*, but can be distinguished by the following characters: Integumental ridges relatively narrower and markedly raised (integumental ridges thick, and not markedly raised in *A. japonicus*); peripheral integumental ridges narrower and more elongate, and regularly arranged (peripheral ridges thick and short, and irregularly arranged in *A. japonicus*); hypostome of female extending to mid-length of palpal article 3 (extending to mid-length of palpal article 2 in *A. japonicus*); article 3 shorter than article 4 (article 3 equal to article 4 in *A. japonicus*); each tarsus of nymph with a prominent dorsal subapical protuberance (no dorsal subapical protuberance in *A. japonicus*) (45).

### *Argas beijingensis* Teng, 1983

This species was first described in Beijing, China (55). The specific name *beijingensis* is derived from “Beijing,” China, the origin of the type species, plus the Latin adjectival suffix “-ensis,” meaning “belonging to.”

#### Type depositories

Institute of Zoology, Chinese Academy of Sciences (IZAS) (holotype ♀, allotype ♂, paratypes 3♀♀ 2♂♂ 4 nymphs and 4 larvae).

#### Local distribution

Palearctic Region (Beijing, Hebei, Shandong) (51–55).

#### Natural host

Columbiformes: pigeon (*Columba livia*), *Streptopelia chinensis*; Passeriformes: sparrow (*Passer montanus*), swallow (*Hirundo rustica*); Galliformes: chicken (*Gallus gallus domesticus*) (51–55).

#### Habitats

Avian nests and their surroundings.

#### Molecular data

No record.

#### Tick-borne pathogens

No record from China.

#### Remarks

According to Teng (55), this species is closely related to *A. reflexus*, but can be distinguished by the following characters: In adults, body slightly broader posteriorly (markedly broadened in posterior one-third in *A. reflexus*), the fixed digit of chelicera with two teeth (three teeth in *A. reflexus*), the setae on tarsi I–IV different in number between *A. beijingensis* and *A. reflexus*; in larva, body oval with an approximate oval plate of the dorsum (body subcircular with a relatively narrower and longer plate in *A. reflexus*), eight pairs of seta in posterolateral quadrants of the dorsum (nine pairs in *A. reflexus*). *Argas beijingensis* is also related to *A. vulgaris*, but can be distinguished by the following characters: In adults, the anus slightly posterior to the center of venter (much more separated from the middle of ventral body surface in *A. vulgaris*), peripheral integumental ridges short and sinuous (relatively narrower and longer in *A. vulgaris*); in larva, body oval, and its dorsolateral margin with 24–25 pairs of setae (body subcircular, and its dorsolateral margin with 19–21 pairs of setae in *A. vulgaris*) (55).

Sun et al. (53) reported specimens from Inner Mongolia, Beijing, Hebei, Shanxi, Shandong, Shaanxi, Jiangsu, Shanghai, Anhui, Fujian, Taiwan, and Sichuan in China as *A. beijingensis*, which were initially recorded as *A. reflexus*. However, according to the descriptions of Sun et al. (53), they only checked the specimens from Shandong; other specimens were not re-examined, thus the distribution of *A. beijingensis* should be further investigated.

### *Argas japonicus* Yamaguti, Clifford & Tipton, 1968

#### Local distribution

Palearctic Region (Beijing, Hebei, Jilin, Liaoning, Inner Mongolia, Ningxia, Xinjiang); Oriental Region (Taiwan) (33, 51–58).

This species has also been reported in Japan and Korea (59), and has been studied more in depth in Japan.

#### Natural host

Columbiformes: *Streptopelia* spp.; Passeriformes: swallow (*Hirundo daurica japonica, Deliclion dasypus*), sparrow (*Passer* spp.); Galliformes: chicken (*Gallus gallus domesticus*) (33, 51, 53–58).

It has been reported that the overwhelming majority of specimens have been collected from swallows and swallow nests (54, 60–69). Researchers rarely collected *A. japonicus* ticks from hosts other than wild birds, although it has been found that this species successfully sucks blood from chickens and many mammals in the laboratory (62, 64). Zhao et al. (56) first reported *A. japonicus* collected from cattle in nature, and found this species actively infesting livestock from February to March in spring in Xinjiang. They also screened the pathogens of fed *A. japonicus* ticks from cattle and found spotted fever group *Rickettsia* spp. and “*Candidatus* Anaplasma boleense” in this species (56). In Japan, Uchikawa (62) used chicken skin as a feeding membrane to study feeding behavior of *A. japonicus*. The results indicated that most *A. japonicus* ticks fed on chicken, rabbit, sheep, and bovine blood could develop successfully but those fed on human, horse, and pig blood showed high mortality rates (68–77%) (62). However, the reason for this difference remains unknown. Several human infestations by this species have been reported in China and Japan (33, 57, 70). In China, the first reported case of human dermatitis caused by *A. japonicus* biting was recorded in Liaoning, China in 1986 (34). In April 2016, several human cases of *A. japonicus* ticks biting were reported in Inner Mongolia Autonomous Region of China, and the patients appeared to have fever, skin rash, swelling, itching and inoculation eschars (57). Subsequently, the microbiota of free-living *A. japonicus* in the affected community was explored (57). In Japan, a group of elderly patients with physical disabilities experienced infestation with *A. japonicus* coming from sparrow nests located under the eaves of a rehabilitation hospital. The tick bites were painful and accompanied by pruritus (70). Therefore, *A. japonicus* selects birds, especially swallows, as its primary and preferred hosts. Human and other mammals may act as accidental hosts.

#### Habitats

This species often inhabits the nests of birds, occasionally hencoops, poultry and livestock yards, and attacks people at night.

#### Molecular data

China: 16S rDNA (MH782636), 12S rDNA (MG668793– MG668795).

#### Other countries

Japan: 16S rDNA (AB819156, AB819157), mitochondrial genome (MT371799).

#### Tick-borne pathogens

Spotted fever group rickettsiae (56, 57), *Alcaligenes faecalis* (57), “*Candidatus* Anaplasma boleense” (56).

There are a few studies on the pathogens and diseases transmitted by *A*. *japonicus*. Further investigations of *A. japonicus* and its pathogens should be conducted in China.

### *Argas persicus* (Oken, 1818)

#### Local distribution

Palearctic Region (Beijing, Xinjiang, Gansu, Qinghai, Hebei, Jilin, Liaoning, Heilongjiang, Inner Mongolia, Shandong, Shanxi, Shaanxi); Oriental (Shanghai, Hubei, Fujian); Paleozoic–Oriental ecotone (Anhui, Sichuan, Jiangsu) (17, 52–54, 71–76).

#### Natural host

Columbiformes: pigeon (*Columba* spp.); Passeriformes: sparrow (*Passer monatanus*), swallow (*Hirundo* spp.); Galliformes: chicken (*Gallus gallus domesticus*) (17, 52–54, 71–76).

This species appears to be mainly a parasite of domestic fowl and arboreal nesting birds (77). It commonly attacks humans, causing it to have an evil reputation especially in early Persia (77). Additionally, this species can sometimes be found in domestic animals, especially sheep and cattle, in China (51), which has not been reported in other countries or areas (77–83). This is mainly because domestic animals are often mixed and housed together with poultry in the rural areas of China.

#### Habitats

In the crevices of poultry houses and nearby human houses, or in the cracks or under the bark of trees frequented by their wild avian hosts.

#### Life cycle

Qi et al. (31) and Tian (30) thoroughly studied the life history of *A. persicus* in a laboratory. Di (39, 43) reported on its life habits and the seasonal and diurnal activities. In Shandong, *A. persicus* was found from early March to mid-October with an active period from May to September and peak prevalence in July (75). The overwintering period for this species was from November to February. This species endures for long periods, with larvae starving up to 8 months, nymphs 24 months and 3 years in adults. Any developed stage of *A. persicus* could overwinter, and the longevity was reported to be 10–20 years (43). The activity of *A. persicus* larvae was not limited by day and night. In contrast, the activity of *A. persicus* nymphs and adults was affected by light and mostly were active at night. Usually, the larva is attached to the featherless part or near the feather roots of poultry, where they can suck blood, whereas the nymphs and adults are attached to the featherless toes of poultry. There were two to seven instars in the nymphal stage with similar morphological features, but they increased in size. Ticks began seeking for hosts at 7–8 pm, reaching a peak around midnight from late July to mid-September (39). The mating behavior between males and females was carried out after sucking a small amount of blood during the day or night (30). Ticks only climbed to the host when sucking blood and left the host immediately after completion of the blood meal. Most of the larvae usually fed for 2–7 days, while very few fed for 10 days. Each nymphal instar fed for several minutes to several hours, and the adults were generally replete in 15 min to 3 h. The molting period of larva was 6–18 days (30) or 4–17days (31), while the molting time of first instar nymph was 7–12 days (30) or 10–97 days (31), and that of second instar nymph was 9 days (30) or 12–63 days (31) under 26–28°C with 65–85% relative humidity. The various molting period of nymph may be related to individual differences and blood engorgement levels (30, 31).

According to the experimental observations, the preoviposition stage of females has been reported to range from 3 to 160 days (30, 31). Oviposition time seems to be related to the month in which females are fed. The preoviposition period of females sucking blood from June to August was the shortest, whereas that of females sucking blood in January was the longest. They generally oviposited in 4–21 days after engorgement, and the number of eggs was related to the amount of bloodsucking. Generally, 50–200 eggs were laid at a time, and more than 1,000 eggs could be laid in the lifetime of a female.

#### Molecular data

China: 16S rDNA (MN894073, MK555333, KR297208, KR297209, LC209197, LC209198, KX258880); COI (LC209195, LC209196, MN900726, MK571448); mitochondrial genome (OM368319, OM368320, MT012684, NC_053794).

#### Other countries

Australia: 16S rDNA (AY436769, AY436770, AY436772); Egypt: 16S rDNA (AF001402); COI (OM177661); Iran: Cathepsin L-like protein (MN175238, MN175239); COI (KX879770); Italy: 16S rDNA (GU451248); Kazakhstan: COI (MN900726); Kenya: 28S rDNA (KJ133607); 18S rDNA (KJ133633); ITS1 (KJ133633); ITS2 (KJ133607); mitochondrial genome (KJ133581); Pakistan: 16S rDNA (MZ496987, MT002847); Romania: COI (FN394341); NAD5 (FN394358); South Africa: 16S rDNA (GU355920); USA: 18S rDNA (L76353); 16S rDNA (L34321); 12S rDNA (GU355920); COI (U95864).

#### Tick-borne pathogens

*Borrelia anserina, Francisella tularensis, Coxiella burnetii, Rickettsia hoogstraalii, Coxiella*-like endosymbiont, *Pseudomonas geniculata, Sphingomonas koreensis, Acinetobacter haemolyticus, Streptococcus suis, Staphylococcus aureus* (84–87).

In China, *A. persicus* has been reported to carry many pathogens, as described above, and only *B. anserina* is well-known to cause fowl spirochetosis. There are many cases of illness in chickens, geese, and ducks bitten by *A. persicus* in China (24–27, 32, 72, 87). In 2006, there was an outbreak of goose spirochetosis in Inner Mongolia, which caused mortality in nearly half of the sick geese, and many adults of *A. persicus* were found in goose housings. Clinical symptoms, pathological anatomy, and microscopic examination indicated that the goose disease was caused by *B. anserina*, transmitted by the vector *A. persicus* (87). Additionally, it was also reported that most chickens lost their appetite, were emaciated, and even died within a week in Gansu China, because of the infestation by *A. persicus* larvae. The chickens were observed for depression, fluffy feathers, liquid stools, crowns, beards, feet visible mucous membranes of pale color, and unstable standing or paralysis. However, the cause of the disease or pathogens has not yet been reported (71).

In other countries or regions, this species has also been reported to transmit *Aegyptionella pullorum* (Aegyptianellosis), Slovakia virus, Kyasanur forest disease virus and *Francisella persica*, which all have not been detected in China (88–92).

#### Remarks

*Argas persicus* is considered native to Turanian–Central Asia but with human activities it become established throughout most continents except Antarctica (78). Many records report the presence of this species in Taiwan (51, 54, 72). However, Robbins (93) believed that published references to *A. persicus* in Taiwan were misidentifications (93–98). Indeed, *A. persicus* listed in Taiwan by Teng (72) may represent the morphologically similar *A. robertsi* (93). Thus, records of *A. persicus* from the Oriental region should be further determined.

Zhou and Meng (99) studied the karyotypes of 56 *A. persicus* ticks and found 51 ticks were diploids, i.e., 2*n* = 26 (24 + XY) (♂); 2*n* = 26 (24 + XX) (♀). Interestingly, it was also discovered that four ticks were tetraploid (4*n* = 52) and one tick was octoploid, i.e., 8*n* = 104 (96 + XXXXYYYY). Zhou and Meng (99) speculated that the reason of this polyploidy could be related to the use of colchicine during the sample processing (99). Additionally, they also reported that the Y-chromosome of *A. persicus* from Xinjiang was 37.8% the length of the X-chromosome, and the average length of all autosomes was 14.7% the length of the X-chromosome (99). Goroschenko (100) reported those ratios of *A. persicus* from the former USSR as 54.4 and 26.5%, respectively. These differences might be related to tick strains from different geographical areas.

### *Argas pusillus* Kohls, 1950

#### Local distribution

Oriental (Taiwan) (51–53, 93).

*Argas pusillus* is a typical southeastern Asian species that has been reported in Philippines, China, Thailand, Malaysia, and Singapore (5, 51, 93, 101–104).

#### Natural host

Chiroptera: bats (*Scotophilus temminckii, Pipistrellus imbircatus*).

This species mainly parasitizes bats, specifically *Scotophilus* spp.

#### Habitats

Near bat caves.

#### Molecular data

No record.

#### Tick-borne pathogens

No record from China.

Studies on *A. pusillus* and its pathogens all over the world are very limited, mainly including species examination, distribution, hosts and a few on pathogen detections (101–106). To date, Issyk–Kul fever virus and Keterah virus have been reported in this species (5, 105, 106).

#### Remarks

It is often confused with the bat tick, *A. vespertilionis*. Hoogstraal (letter No. 251, February 14, 1984 and letter No. 376, February 14, 1977) concluded that the samples of *A. vespertilionis* collected in Taiwan were *A. pusillus* (93). Robbins (93) stated that the published records of *A. vespertilionis* in Taiwan (72, 94, 95, 98, 107) may represent *A. pusillus* (93). In addition to Taiwan, this species probably also occurs in other areas of China; therefore, *A. vespertilionis* collected from southern China should be further re-examined.

### *Argas reflexus* (Fabricius, 1794)

#### Local distribution

Palearctic (Gansu, Qinghai, Hebei, Henan, Inner Mongolia, Ningxia, Shandong, Shaanxi, Xinjiang, and Heilongjiang); Palaeozoic–Oriental ecotone (Anhui).

*Argas reflexus* can be found in the Palearctic region between parallels 31°N and 51°N (108, 109). This species is widely distributed in Europe and has been reported in some regions of Asia (Israel, Turkey, Iran, Pakistan, Afghanistan and Kazakhstan), as described in detail by Pfäffle and Petney (108). Additionally, Hoogstraal and Kohls (110) found a single unengorged larva of *A. reflexus* in Egypt.

#### Natural host

Columbiformes: pigeon (*Columba livia domestica, Columba rupestris*); Passeriformes: sparrow (*Passer* spp.), swallow (*Hirundo* spp.), chough (*Pyrrhocorax graculus*); Galliformes: chicken (*Gallus gallus domesticus*) (19, 22, 23, 42, 51, 53, 55, 73, 111, 112).

*Argas reflexus* predominantly parasitizes domestic pigeons (*Columba livia domestica*) and bites other birds, including rock pigeons (*Columba livia*), rock swallow (*Ptyonoprogne rupestris*), turtle doves (*Streptopelia turtur*), fan–tailed ravens (*Corvus rhipidurus*), jackdaw (*Corvus monedula*), swifts, swallows, owls, crows, several passerine birds, chickens and even humans (108–110, 113).

#### Habitats

Inhabit pigeon and other bird nests, and the vicinity of its hosts.

#### Molecular data

China: No record.

#### Other countries

Luxembourg: arg-r-1 (AJ697694); Poland: 16S rDNA (AF001401); Spain: 16S rDNA (MW289075, MW289076, MW289084); COI (MW288388); USA: 16S rDNA (L34322); 12S rDNA (U95865).

#### Tick-borne pathogens

No record from China.

It has been reported that *A. reflexus* is a vector of *Aegyptianella pullorum*, Crimean–Congo hemorrhagic fever virus, Uukuniemi virus, Grand Arbaud virus, Ponteves virus, Tunis virus, West Nile virus, Chenuda virus, Nyamanini virus, and Quaranfil virus (98, 114–118).

#### Remarks

Teng (55) concluded that *A. reflexus* published in “Economic Insect Fauna of China Fasc 15” was misidentified: specimens collected from Xinjiang should be *A. vulgaris* while those collected from Beijing should be *A. beijingensis*. However, he did not mention the specimens collected from other regions. Yu et al. (73) reported this species in Xinjiang. Based on the geographical location of China and the distribution area and host characters of *A. reflexus*, it is possible for this tick species to appear in China. Therefore, this species has been kept in the valid tick list of China until conclusive evidence is obtained.

### *Argas robertsi* Hoogstraal, Kaiser & Kohls, 1968

#### Local distribution

Oriental (Taiwan) (52–54, 93).

*Argas robertsi* is common in Australia (Queensland, Northern Territory, New South Wales) and the Indo–Malaya region, including Indonesia (Java), China (Taiwan), Thailand, India (West Bengal), and Sri Lanka (82, 119, 120).

#### Natural host

Galliformes: chicken (*Gallus gallus domesticus*), Pelecaniformes: cormorant (*Phalacrocorax* spp.), ibis (*Threskiornis* spp.); Ciconiiformes: heron (*Ardea* spp., *Ardeola* spp., *Bubulcus* spp., *Nycticorax* spp., *Egretta* spp., *Plegadis* spp.), stork (*Anastomus* spp.) (29).

#### Habitats

Often inhabits bird nests, occasionally occur in hencoops.

#### Life cycle

Hoogstraal et al. (119) studied the life cycle of *A. robertsi* collected from Taiwan, using domestic pigeons as experimental hosts at 28–30°C and 75% RH. The life cycle of *A. robertsi* was 2–10 months and included two to five nymphal instars in Taiwan, similar to other *A. robertsi* populations from different regions. The nymphs and adults fed within a few days of molting. Many males molted from the earlier nymphal instars. Most females needed to suck blood twice to lay eggs, while few needed to suck blood only once (119).

#### Molecular data

China: No record.

#### Other countries

Australia: 16S rDNA (AY436768).

#### Tick-borne pathogens

Kuo Shuun virus (29).

Other viruses, including CSIRO 1499 virus, Lake Clarendon virus, Nyamanini virus and Pathum Thani virus have also been detected in other countries or regions (82, 105).

#### Remarks

Barker and Walker (82) stated that *A. robertsi* and *A. persicus* lived in sympatry in Australia. Although *A. persicus* is very common in China, *A. robertsi* has only been reported in Taiwan. Further investigations on *A. robertsi* and *A. persicus* should be conducted in China.

### *Argas sinensis* Jeu & Zhu, 1982

The specific name “*sinensis”* means “belonging to China.”

#### Type depositories

Department of Parasitology, Chongqing Medical College, Chongqing, China (holotype one unfed larva, paratypes two unfed larvae, two partly engorged larvae, and four engorged larvae).

#### Local distribution

Oriental (Sichuan) (36, 38, 52).

#### Natural host

Chiroptera: bat (*Pipistrellus abramus*).

Jeu (36) stated that larvae could feed successfully on white rats and mice. Nymphs and adults could feed well on a wide range of vertebrate animals (including *Rattus tanezumi, Rattus norvegicus, Mus musculus*, guinea pig, rabbit, dog, cat and monkey) and poultry (including chicken, goose, duck, and pigeon) under laboratory conditions. Jeu even contributed his skin to verify that humans are also suitable hosts for ticks (36).

#### Habitats

Occurs in bat colonies, often can be found in bat infested buildings (38).

#### Life cycle

Jeu (36) carefully investigated the life history of *A. sinensis* collected from Chongqing, under laboratory conditions from 1973 to 1977 (36). There were two to four nymphal instars for this species. The molting nymph could be divided into the following three types: (1) composed of two instars that sucked blood twice; (2) composed of three instars that sucked blood 3 times; and (3) composed of four instars, the first instar nymph could molt into the second instar nymph directly without sucking blood, then sucked blood 3 times. Females laid eggs several times, with prolonged oviposition periods, but delaying the time between oviposition periods progressively. They were able to deposit four to eight batches of eggs, totaling 144–423 eggs (36).

#### Molecular data

No record.

#### Tick-borne pathogens

No record from China.

#### Remarks

The larva of this species is closely related to *A. vespertilionis* and *A. daviesi*, but differs from them in the following characters: (1) dorsal setae numbering 14 pairs; (2) body with 11 pairs of dorsoexternal setae and micro setae; and (3) relative distance between postpalpal and posthypostomal setae 2.3:1 (38).

### *Argas vespertilionis* (Latreille, 1796)

#### Local distribution

Palearctic (Hebei, Shandong, Henan, Gansu, and Xinjiang); Oriental (Hubei, Hunan, Guangdong, Zhejiang, Guizhou, Fujian, Guangxi, and Yunnan); Paleozoic–Oriental ecotone (Sichuan, Jiangsu) (37, 51–53, 55, 121, 122).

#### Natural host

Chiroptera: bat (*Vespertilio* spp.) (37, 51–53, 55, 121, 122).

This species parasitizes bats, and occasionally attacks humans.

#### Habitats

Associated with bats and bat habitats.

#### Molecular data

China: 16S rDNA (MW132811, MF106219–MF106221, KY657240, OK047498, OK054512); COI (KY657239); mitochondrial genome (OM368317, OM368318).

#### Other countries

Belgium: COI (MK140084, MK140088); France: 12S rDNA (JX233821); Hungary: 16S rDNA (KX831484–KX831489); COI (KX431953–KX431955); Italy: 16S rDNA (KX831496–KX831498, HM751841); Japan: 16S rDNA (AB819158); mitochondrial genome (MT762370); Kenya: 16S rDNA (KX831491); COI (KX431956); Netherlands: COI (MK140082, MK140083, MK140085–MK140087); Pakistan: 16S rDNA (MK571555); COI (MK571553); Romania: 16S rDNA (KX831490); Spain: 28S rDNA(MT739330, MT739331); 18S rDNA(MT739410, MT739411); 5.8S rDNA(MT739330, MT739331); ITS1(MT739410, MT739411, MT739330, MT739331); ITS2(MT739330, MT739331); mitochondrial genome (MT680027, MT680028, NC_060373); United Kingdom: 16S rDNA (MF510175–MF510177); COI (MF510173, MF510174); Viet Nam: 16S rDNA (KX831492–KX831495); COI (KX431957–KX431960).

#### Tick-borne pathogens

*Babesia vesperuginis, Rickettsia raoultii, Rickettsia rickettsia* (121, 123, 124).

In other parts of the world, this tick species has been reported as a vector of Issyk–Kul, Keterah, and Sokuluk viruses, Q fever rickettsia, *Coxiella burnetii, Ehrlichia* sp. AvBat, *Rickettsia* sp. AvBat, *Borrelia burgdorferi sensu lato* and an unknown *Borrelia* species closely related to *B. recurrentis, B. crocidurae* and *B. duttonii* (101, 125).

#### Remarks

*Argas vespertilionis* is confused with morphologically similar species, therefore, the global distribution of this species is not clear. It appears that *A. vespertilionis* is widely distributed in Africa, Europe, the Palearctic parts of Asia, and a few parts of the oriental region, including some parts of India, Cambodia (126), Vietnam (123) and southern China (5, 51, 52, 93). Hoogstraal (5) stated that reports of *A. vespertilionis* from other parts of the oriental region (Bangladesh, Malaysia, and Philippines) were misidentifications with *A. pusillus*. Robbins (93) excluded *A. vespertilionis* from the checklist of tick species in Taiwan and corrected it to *A. pusillus*. Then, identifications of *A. pusillus* in China are all from Taiwan, and those oriental records of the *A. vespertilionis* are currently doubtful. Therefore, the occurrence of these two species in China should be reconsidered.

### *Argas vulgaris* Filippova, 1961

#### Local distribution

Palearctic (Xinjiang, Jilin, Gansu, Ningxia, Liaoning, Beijing, Hebei, Inner Mongolia, Shanxi, Shandong, Shaanxi) (51–53, 55).

Filippova (8) indicated that this species was widely distributed in the Palearctic region and was common in the former Soviet Union.

#### Natural host

Columbiformes: pigeon (*Columba* spp.); Passeriformes: sparrow (*Passer* spp.) (51–53, 55).

#### Habitats

Often inhabits bird nests.

This species inhabits lowland and foothill meadow steppes, dry steppes, and deserts. Its vertical distribution ranges from sea level (lower reaches of the Talghinka River in Dagestan) to 900 m above sea level (Karabil, Turkmenistan). Its favorite habitats are ground nests or burrows of birds in outcrops of loess, sandstone, and limestone, as well as the steep banks of rivers and lakes (8).

#### Molecular data

China: No record.

#### Other countries

Poland: 16S rDNA (AF001404).

#### Tick-borne pathogens

No record from China.

Few studies have been conducted on the pathogens of *A. vulgaris*. Hissar virus (*Bunyaviridae*) and Tyulek virus (*Orthomyxoviridae*) were isolated from this tick species in Tadjikistan and Kyrgyzstan, respectively (127, 128).

#### Remarks

Teng (55) stated that *A. reflexus* from Xinjiang published by Teng (72) should be *A. vulgaris*. Yu et al. (73) reported only *A. reflexus* in Xinjiang. In terms of geographic location and climate, both species have the potential to be distributed in Xinjiang. Therefore, the tick specimens of Xinjiang need to be re-examined.

### *Ornithodoros capensis* (Neumann, 1901)

#### Local distribution

Oriental (Taiwan) (93).

This species is globally distributed along the coasts and islands of the Pacific, Atlantic and Indian Oceans; the Caribbean and Coral Seas and the lakes of the eastern African Rift Valley system (5, 129–131). Except for Taiwan, very few surveys have been conducted in other parts of China along the coastline, especially in the southern part where the species might also be distributed.

#### Natural host

No record from China.

#### Habitats

Inhabits in seabird nests.

#### Molecular data

China: No record.

#### Other countries

Algeria: 16S rDNA (KP776644); Australia: 16S rDNA (AH011497); COI (AH011497); NAD1 (AH011497); Brazil: 16S rDNA (KU757069); Cape Verde: 18S rDNA (JQ824327–JQ824368); 16S rDNA (JQ824295–JQ824326); Japan: 16S rDNA (AB819266, AB242431, AB242431, AB057537–AB057540, AB076080–AB076082); mitochondrial genome (AB075953, NC005291); USA: 16S rDNA (EF636462, EF636466).

#### Tick-borne pathogens

No record from China.

It has been reported that this species can transmit Soldado virus, West Nile virus, Johnston Atoll virus, Upolu virus, Nyaminini virus, Quaranfil virus, Saumarez Reef virus, Hughes virus, *Rickettsia* spp. and *Borrelia* spp. (129, 132).

#### Remarks

Although China has many islands scattered along the seashore, studies on seabird ticks are scarce, with the exception of *O. capensis*. It is known that both seabird ticks *O. sawaii* and *O. maritimus* are distributed in Palearctic region. *O. maritimus* is distributed in Great Britain, Ireland, France (Corsica), Tunisia, Portugal, Italy (off Sardinia), southwestern USSR, and Senegal (133). *Ornithodoros sawaii* is reported from Republic of Korea and Japan (133, 134). Therefore, these two species might also be distributed in the islands of China.

### *Ornithodoros huajianensis* Sun, Xu, Liu & Wu, 2019

The specific epithet is in allusion to the habitat where this species was found (16).

#### Type depositories

Medical Entomology Gallery of Academy of Military Medical Sciences, Beijing, China (AMMSC) (holotype ♀, paratypes 2♀♀ 3♂♂ and 3 nymphs).

#### Local distribution

Palearctic (Gansu) (16).

#### Natural host

Rodentia: *Marmota bobak sibirica* (16).

#### Habitats

Prefer semiarid hilly steppes.

#### Molecular data

China: 16S rDNA (MK208992–MK208994).

#### Tick-borne pathogens

No record.

#### Remarks

This species belongs to the subgenus *Ornithodoros*. It was diagnosed by its broad rectangular tongue and triangular tongue–shaped posterior lip in the male genital apron, a shallow camerostome with definite folds, and smaller mammillae with a single seta mixed with larger ones in nymphs and adults (16).

### *Ornithodoros lahorensis* (Neumann, 1908)

#### Local distribution

Palearctic (Xinjiang, Inner Mongolia, Shandong, Gansu, Liaoning, and Tibet) (18, 20, 41, 51–53, 56, 72, 135, 136).

This species is widely distributed in the Palearctic region, including Armenia, Dagestan, Kazakhstan, Uzbekistan, Turkmenistan, Kyrgyzstan, Tajikistan, Russia, Kosovo, Republic of Macedonia, Syria, Turkey, Iran, Iraq, Saudi Arabia, Afghanistan, Lebanon, Syria, Pakistan, Bulgaria, Greece, Israel, China, and India (5, 8, 132, 137–141).

#### Natural host

Carnivora: dog (*Canis* spp.); Artiodactyla: cattle (*Bos* spp.), sheep (*Ovis* spp.), goat (*Capra* spp.), camel (*Camelus* spp.); Perissodactyla: horse (*Equus* spp.) (18, 20, 41, 51–53, 56, 72, 135, 136).

This species was originally as a parasite of the Asiatic mouflon, *Ovis orientalis arkal*, and other wandering ungulates resting beside cliffs. However, nowadays, it is a notorious parasite of sheep, camels, and cattle, especially in primitive stables and dwellings in steppes and mountain deserts (5). This species has also been reported to infest human in Turkey and the former Soviet Union (138–140).

#### Habitats

Living mainly in sheep pens or other livestock sheds (also found in chicken coops). It is rarely reported from natural habitats.

#### Molecular data

China: 18S rDNA (KX530878, KX530879); 16S rDNA (MG651950–MG651959, KX530872–KX530877, ON159478–ON159502, MN564903–MN564909, OM673115–OM673125, OL444952–OL444957); 12S rDNA (MG651960–MG651967); COI (KX530866–KX530871).

#### Other countries

Afghanistan: 18S rDNA (L76354); Iran: COI (MK318148, MG582607).

#### Tick-borne pathogens

“*Candidatus* Anaplasma boleense” and *Anaplasma ovis* (35, 56).

Other pathogenic associations include Crimean–Congo haemorrhagic fever (CCHF) virus, *Rickettsia sibirica, R. conorii, Brucella abortus, F. tularensis*, and *C. burnetii*, which have not been detected in China (139).

#### Life cycle

*Ornithodoros lahorensis* is one of the most studied species of soft tick in China. Shao (28) studied the biology of *O. lahorensis* feeding on rabbit under laboratory conditions in Xinjiang. After hatching, it took more than one month for larvae at room temperature before they were able to attach to a host, and then took a total of 24–42 days for blood–sucking larvae to become engorged third instar nymphs (28). Engorged third instar nymphs molted into males and females for 113–149 days and 110–147 days, respectively. Newly molted adults needed 1–1.5 months before attaching to hosts. Engorged females laid eggs between June to August, peaking in July. In Xinjiang, adults and third-instar nymphs could overwinter in the wall crevices of a sheep fold. The larvae infested sheep in late September and October. Zhao et al. (56) reported that *O. lahorensis* ticks infested livestock from late February to early April in southern Xinjiang.

### *Ornithodoros papillipes* (Birula, 1895)

#### Local distribution

Palearctic (Shanxi, Xinjiang, Inner Mongolia, and Shaanxi) (40, 44, 48, 51, 53, 72).

The species is widely distributed in the Mediterranean and Central Asian subregions of the Palearctic, including Kazakhstan, Uzbekistan, Turkmenistan, Kyrgyzstan, Tajikistan, eastern Libya, western Egypt, Turkey, Cyprus, Syria, Lebanon, Israel, Early Jordan, Iraq, Saudi Arabia, Iran, Afghanistan, Pakistan (Kashmir and western Punjab), and China (8). However, owing to confusion in systematics, some of these data require clarification (8).

#### Natural host

Carnivora: dog (*Canis* spp.), fox (*Vulpes* spp.); Artiodactyla: sheep (*Ovis* spp.); Lagomorpha: hare (*Lepus* spp.); Erinaceomorpha: hedgehog (*Erinaceus* spp.); Soricomorpha: scilly shrew (*Crocidura suaveolens*); Anura: toad (*Bufo viridis*) (40, 44, 48, 51, 53, 72).

#### Habitats

It usually selects caves, grottoes, and burrows inhabited by small and medium-sized animals in desert and semi-desert areas along its distribution. In some regions, it often occurs in livestock stables and human houses.

#### Life cycle

In China, many studies on the biology of *O. papillipes* have been carried out by early researchers (48), which will be very important for distinguishing *O. papillipes* from *O. tholozani*. Engorged females oviposit eggs in summer and autumn (48). Feng et al. (48) reported that there were three to six nymphal instars for this species using mice (*Mus musculus*) and guinea pigs (*Cavia porcellus*) as hosts. A few engorged third instar nymphs molted to adults with the number of males > females; most engorged fourth instar nymphs molted to adults with the number of females > males; a few engorged fifth instar nymphs molted to adults and very few fifth instar nymphs still molted to sixth instar nymphs. The whole process from egg to adult took 5 months to 1 year, which was determined by external temperature and other conditions (48). Additionally, guinea pig (*Cavia porcellus*), chicken (*Gallus gallus domesticus*) and grassland tortoise (*Testudo horsfieldii*) were used as hosts. The results showed that tick development was different under the same laboratory conditions. According to the average weight and volume of engorged ticks, guinea pig is the best host, followed by chicken and then turtle (48).

#### Molecular data

China: No record.

Czech: Defensin (FJ222575–FJ222577).

#### Tick-borne pathogens

*Borrelia persica* (40).

In the 1950–1980s, many cases of tick-borne relapsing fever were reported in Xinjiang. In southern part of this province, the pathogen was *Borrelia persica* transmitted by *O. papillipes* (40, 48, 142). Feng et al. (48) stated that the natural infection rate of spirochetes was very high in *O. papillipes* with spirochetes isolated from 12 of 13 tick groups collected from wall crevices of human houses and burrows of *Bufo viridis*. Additionally, the authors collected many *Bufo viridis* from the same habitats as *O. papillipes*. They then dissected the internal organs (liver, spleen, etc.) of *Bufo viridis*, prepared a suspension emulsion with normal saline, and injected intraperitoneally into guinea pigs. Spirochetes were found in the blood of guinea pigs, which proved that *Bufo viridis* was the natural carrier of tick-borne relapsing fever pathogen (48). Another clinical experiment indicated that 13 guinea pigs suffered from relapsing fever after being bitten by naturally infected *O. papillipes* ticks (80–150 ticks per guinea pig). The incubation period was 4 to 6 days, and the course of the disease lasted 15–20 days. Spirochetes appeared in large numbers in the peripheral blood of these animals. On average, more than 20 spirochetes were observed per field in thick blood smears and in some cases, they were so abundant that could not be reliably counted. During the course of the disease, two guinea pigs died when a large number of spirochetes appeared (48). Shao (40) stated that *O. papillipes* is in close contact with human beings in Xinjiang. They surveyed 50 households in a village and found 49 households were infested by this species. Therefore, in the 1980s, the harm caused by tick-borne relapsing fever in Xinjiang was notable.

Filippova (8) stated that *O. papillipes* was the main vector of tick-borne relapsing fever in the republics of Central Asia and Kazakhstan as well as in neighboring foreign countries. By testing spontaneous carriage, experimental infection and the precipitation reaction a wide range of wild, domestic, and farm animals, carriers of spirochetes in natural and village foci have been established. However, some domestic animals, such as sheep and goats, were characterized by low spirochetemia, resulting in these animals serving only as secondary sources of spirochetes (8). Ticks are capable of taking up spirochetes at any phase and stage, and transmitting them both transstadial and transovarial. The bite of a single infected tick is sufficient to infect humans with spirochetosis (8).

Under experimental conditions, *O. papillipes* can acquire *C. burnetii*, store it for a long time period, transmit the pathogen transstadially, and infect healthy animals during subsequent feeding (8).

#### Remarks

This species is considered a synonym of *O. tholozani* (Laboulbène and Mégnin, 1882) by Neumann (143, 144), which was subsequently accepted by many Western scientists (3, 5). Currently, *O. tholozani* is reported from India, Kazakhstan, Kyrgyzstan, Tajikistan, Turkmenistan, Uzbekistan, Afghanistan, Iran, Iraq, Syria, Jordan, Turkey, Greece, Israel, Egypt, Cyprus, Libya, and Lebanon (51, 145–147). Nuttall et al. (77) considered *O. papillipes* a dubious species, but noted that Birula's figures were difficult to reconcile with the description of *O. tholozani*, especially with regard to the sides of the camerostome and the tarsi, thus they inserted the original description of *O. papillipes* in their book. Filippova (8) indicated that from the diagnosis and drawings of Laboulbene and Mégnin (1882), it follows that when establishing this species, they had an admixture of species among the type specimens. Indeed, the absenece of cheeks, the structure of the peritremes, hypostome, chelicerae, and legs, as well as larval morphology, suggests that the second species could have been an *Alveonasus* sp. (8). Moreover, Filippova (8) did recognize differences between *O. tholozani* and *O. papillipes*, and thought that Neumann's synonymy relied on the examination of more than one species, likely *O. tholozani* and *O. lahorensis*. She also pointed out that in the literature the species *O. tholozani* should be morphologically similar species *O. papillipes, O. verrucosus, O. lahorensis*, and other West Asian species (8). In Russian literature, the most common name is *papillipes*. Therefore, Eastern European workers strongly defend the validity of the name *O. papillipes* with scientifically sound arguments. Guglielmone et al. (3) pointed out that the uncertain status of these taxa led them to treat *O. tholozani* and *O. papillipes* both as provisionally valid.

### *Ornithodoros tartakovskyi* Olenev, 1931

#### Local distribution

Palearctic (Xinjiang, Inner Mongolia, and Shaanxi) (40, 48, 51–53, 72).

This species is distributed in the Palearctic region including Kazakhstan, Uzbekistan, Turkmenistan, Kyrgyzstan, Tajikistan, Iran and China (8, 148).

#### Natural host

Rodentia: *Rhombomys opimus*; Testudines: tortoise (*Testudo horsfieldii*) (40, 48, 51–53, 72).

#### Habitats

Mainly inhabit desert and semi-desert areas.

#### Molecular data

China: No record.

#### Other countries

Czech: Defensin (FJ222581, FJ222582).

#### Tick-borne pathogens

*Borrelia latyschewii* (40).

The pathogen *Borrelia latyschewii* is spread by *O. tartakovskyi* in northern Xinjiang of China (40, 48, 142). *Ornithodoros tartakovskyi* plays a much smaller role in the spread of spirochetosis among humans than *O. papillipes* and *O. verrucosus*, due to its confinement almost exclusively to natural habitats, particularly to burrows of small diameter (8). This species also transmits *Coxiella burnetii* and *Acanthocheilonema viteae* (8, 149).

## Conclusions

With the increasing number of new emerging and reemerging tick-borne diseases over the past 20 years, an increasing number of people are paying attention to ticks and tick-borne pathogens. Geographically, China is located in the southeastern part of the vast Eurasian continent, including the Palearctic and Oriental realms and has a variety of ecological types. However, soft ticks and their associated pathogens remain largely unstudied in China. Toward the end of 2021, the argasid tick fauna of China comprised 15 valid species (6.88% of the world's argasid species). Four species are endemic from China: *A. (Argas) assimilis, A. (Argas) beijingensis, A. (Carios) sinensis* and *O. (Ornithodoros) huajianensis*. Although there are currently no reports of these Chinese endemic argasid species in other countries and regions, it is still possible for those species to be distributed in adjacent regions. Except for *O. capensis*, all other *Ornithodoros* species in China are found in the Palearctic region. Except for *A. vulgaris*, which is limited to the Palearctic Region, the greatest number of *Argas* species is present in the Oriental Region or the Oriental + Palearctic Region. *A. persicus* and *O. lahorensis* most often inhabit nearby human houses and commonly attacks people that makes them the two most thoroughly studied argasid ticks in China.

In total, 47 vertebrate species have been recorded as hosts for Argasidae in China. The most commonly reported hosts of soft ticks in China are birds, followed by mammals. Anurans are rare hosts for *O. papillipes*; however, they can harbor infectious relapsing fever *Borrelia* spp. transmitted by this soft tick (48, 51). The fact that amphibians are implicated as reservoirs of relapsing fever spirochetes is interesting, unprecedented in the eco–epidemiology of these agents, and highlights the need to re–study the disease in China. Additionally, *A. japonicus* and *A. persicus* are always reported to infest birds and domestic fowl abroad, while these two species are often found in livestock in China, which might be because domestic animals are often mixed and housed with poultry in Chinese rural areas. Nine species (60%) were recorded parasitizing humans in China (*A. beijingensis, A. japonicus, A. persicus, A. sinensis, A. vespertilionis, A. vulgaris, O. lahorensis, O. tartakovskyi*, and *O. papillipes*). Therefore, soft ticks are no less harmful to humans than hard ticks are.

It is worth noting that some clinical cases have been reported in China. These cases were caused by ticks or tick-borne pathogens such as *A. japonicus, A. persicus, O. lahorensis, O. tartakovskyi*, and *O. papillipes*. However, the pathogens in each case have seldom been investigated. Additionally, molecular research and investigation of soft ticks and their pathogens, especially on species parasitizing birds and bats remains scarce in China. Except for studies on their morphological characters, research in other areas has not been done for *A. assimilis, A. beijingensis, A. pusillus, A. vulgaris, O. capensis, O. tartakovskyi*, and *O. huajianensis* in China. Therefore, it is necessary to carry out comprehensive research on soft ticks and associated pathogens in the future.

## Author contributions

ZC and JL conceived, designed, and drafted the manuscript. Both authors read and approved the submitted manuscript.

## Funding

This review was supported by the Natural Science Foundation of Hebei Province (C20220516), Science Foundation of Hebei Normal University (L2020B17), and Science and Technology Project of Hebei Education Department (QN2020162).

## Conflict of interest

The authors declare that the research was conducted in the absence of any commercial or financial relationships that could be construed as a potential conflict of interest.

## Publisher's note

All claims expressed in this article are solely those of the authors and do not necessarily represent those of their affiliated organizations, or those of the publisher, the editors and the reviewers. Any product that may be evaluated in this article, or claim that may be made by its manufacturer, is not guaranteed or endorsed by the publisher.
